# Evaluation of Toxicity and Neural Uptake In Vitro and In Vivo of Superparamagnetic Iron Oxide Nanoparticles

**DOI:** 10.3390/ijms19092613

**Published:** 2018-09-03

**Authors:** Muhammad Kamran Khalid, Muhammad Asad, Petra Henrich-Noack, Maxim Sokolov, Werner Hintz, Lisa Grigartzik, Enqi Zhang, Alexander Dityatev, Berend van Wachem, Bernhard A. Sabel

**Affiliations:** 1 Institute of Process Engineering, Chair of Mechanical Process Engineering, Otto-von-Guericke University, Universitätsplatz 2, 39106 Magdeburg, Germany; muhammadasad591@gmail.com (M.A.); werner.hintz@ovgu.de (W.H.); berend.vanwachem@ovgu.de (B.v.W.); 2 Institute of Medical Psychology, Otto-von-Guericke University, Leipziger Str. 44, 39120 Magdeburg, Germany; petra.henrich-noack@med.ovgu.de (P.H.-N.); sokol5555@gmail.com (M.S.); lisa.grigartzik@med.ovgu.de (L.G.); enqi.zhang@med.ovgu.de (E.Z.); bernhard.sabel@med.ovgu.de (B.A.S.); 3 German Center for Neurodegenerative Diseases, Leipziger Str. 44, 39120 Magdeburg, Germany; alexander.dityatev@dzne.de; 4 Medical Faculty, Otto-von-Guericke University, Leipziger Str. 44, 39120 Magdeburg, Germany; 5 Center for Behavioral Brain Sciences (CBBS), 39106 Magdeburg, Germany

**Keywords:** superparamagnetic iron oxide nanoparticles (SPIO-NPs), drug delivery, in vitro toxicity, cellular uptake

## Abstract

Superparamagnetic iron oxide nanoparticles (SPIO-NPs) have great potential to be used in different pharmaceutical applications, due to their unique and versatile physical and chemical properties. The aim of this study was to quantify in vitro cytotoxicity of dextran 70,000-coated SPIO-NPs labelled/unlabelled with rhodamine 123, in C6 glioma cells and primary hippocampal neural cells. In addition, we analyzed the in vitro and in vivo cellular uptake of labelled SPIO-NPs. The nanoparticles, with average size of 10–50 nm and polydispersity index of 0.37, were synthesized using Massart’s co-precipitation method. The concentration-dependent cytotoxicity was quantified by using tetrazolium dye 3-(4,5-dimethylthiazol-2-yl)-2,5-diphenyltetrazolium bromide (MTT). Intracellular localization of SPIO-NPs was detected by confocal laser microscopy. In vivo confocal neuroimaging (ICON) was performed on male Wistar rats after intravitreal injection followed by ex vivo retina whole mount analysis. When used for in vitro testing concentrations in the range of diagnostic and therapeutic dosages, SPIO-NPs proved to be non-cytotoxic on C6 glioma cells for up to 24 h incubation time. The hippocampal cell culture also did not show impaired viability at low doses after 24 h incubation. Our results indicate that our dextran-coated SPIO-NPs have the potential for in vivo drug delivery applications.

## 1. Introduction

There is a globally increasing interest in nanoparticles, due to their unique and versatile physical and chemical properties to pass biological barriers [1,2,3]. Especially, particles of 1–100 nm in size with defined characteristics were used in biomedical engineering [4,5] and, according to some reports, nanoscale materials, like metallic nano-gold, have been serving humans for different medical purposes already since 2500 BC [5]. Cobalt, nickel, iron, and iron oxide, such as maghemite (γ-Fe_2_O_3_) and magnetite (Fe_3_O_4_) nanoparticles, have been widely considered as the most suitable materials for medical applications, due to their superparamagnetic property. Among these, iron oxide (along with its different types) is considered as one of the most important metal oxides for technological purposes, and it has the advantage of being widespread in nature. Magnetite (Fe_3_O_4_) is the most prominent among other magnetic oxides, and has been attracting intensive interest in recent years because of its superparamagnetic and electric properties. It is widely used for industrial applications, such as ceramics, catalysts, energy storage, magnetic data storage [6], recording media, pigments, magnetic fluids [7], dyes, in the cosmetic industry for ultraviolet (UV) protection (organic and inorganic submicron UV filters), as organic color filters for liquid-crystal display (LCD) technology, as solar cell constituents [8], in bioengineering, biosensors, magnetic refrigeration, color imaging, bioprocessing, high grade magnetic separation [9], photocatalysis [10], and in lithium-ion batteries and metal chemosensors [11]. Over 500 consumer products containing nanoparticle-related materials are in use today, and this omnipresence increases the likelihood of animals and humans being exposed to nanomaterials—also iron oxide nanoparticles—in the environment.

However, apart from such unintended exposures, there is substantial potential for magnetic nanoparticles (MNPs) in biomedical applications. This relies on the synthesis of high-quality materials, mainly regarding crystallinity and magnetic response. From this perspective, it is essential to minimize the polydispersity and heterogeneity of the particles, and to maximize their magnetic response. For instance, MNPs for drug delivery [12,13,14] and contrast agents for magnetic resonance imaging, must exhibit a high magnetic response to external fields, and should have functionalized, biocompatible surfaces [15].

Following recent advances in nanotechnology, the composition, size, morphology, and surface chemistry of particles can be tailored, which, in combination with their nanoscale magnetic properties, makes them highly attractive for biomedicine. Magnetite (Fe_3_O_4_) nanoparticles are particularly being used in applications such as cell separation, and therapy involving cell labelling and targeting, tissue repair, targeted drug delivery, magnetic resonance imaging (MRI) [16,17], and hyperthermia for cancer treatment [18,19,20]. Due to the highest saturation magnetization, pure metals are highly toxic and extremely sensitive to oxidation; therefore, without suitable surface treatment, such magnetic nanoparticles from pure metals are not suitable for biomedical applications. By contrast, iron oxides are less sensitive to oxidation and, therefore, can give a stable magnetic response [19].

The United States federal drug association (FDA) has already approved 12 nanomedicines containing magnetic iron oxide nanoparticles, mainly for in vivo administration, and a series of new nanosystems are in the pipeline for approval, and even more—including our particles—are in an experimental stage [21,22,23]. Clearly, the toxicity of magnetic nanoparticles is one of the most debatable issues that should be properly investigated. Importantly, nanoparticle toxicity depends on a number of factors like dose, chemical composition, a method of administration, particle size, biodegradability, surface chemistry, and shape [19]. Toxicity also limits the development of further superparamagnetic iron oxide nanoparticles (SPIO-NPs), because knowledge about their toxicity and their penetration into cells and tissues is very limited [24]. A reliable test to evaluate the cytotoxicity of magnetic nanoparticles is the in vitro assays for cell viability using 3-(4,5-dimethylthiazol-2-yl)-2,5-diphenyltetrazolium bromide (MTT), which assesses metabolic activity. However, other techniques are also widely published (for example lactate dehydrogenase (LDH) assays, in vitro hemolysis, gene expression analysis [25,26], etc.).

We have developed dextran 70,000-coated SPIO-NPs for improved biocompatibility, because uncoated SPIO-NPs have several disadvantages regarding medical use, like, for example, a significant tendency for agglomeration, which, in the worst case, may reduce or block blood flow in capillaries after intravenous application. To design coated SPIO-NPs, we chose dextran 70,000 as this molecule was approved for medical use already in 1947, and it is considered one of the safest medicines. In addition, for more flexibility in detecting these SPIO-NPs, it is helpful to trace them by fluorescence imaging techniques. We therefore also labelled the coated SPIO-NPs with a fluorescent dye. As outlined before, NPs toxicity depends on many factors, and can hardly be predicted for individual new NPs formulations. Even though all ingredients for the production of our NPs are non-hazardous, it cannot be excluded that with the resulting nanosystem, new toxic features may appear and induce cell damage. As per our best knowledge, there is still no studies available for cytotoxicity measurements of dextran 70,000-coated SPIO-NPs in C6 glioma cells and primary neural cells. Also, the cytotoxicity of SPIO-NPs labelled with fluorescent dyes has still not been characterized. As this knowledge is a conditional sine qua non for the development of future applications, in this article, we report quantification of toxicity using MTT assay of our fluorescent magnetic iron oxide nanoparticles coated with dextran 70,000. Moreover, results of investigating the cellular uptake of the fluorescently labelled SPIO-NPs, in C6 glioma cells and in vivo after intravitreal injection, are presented.

## 2. Results and Discussion

### 2.1. Nanoparticle Size and Size Distribution

**Figure 1:**
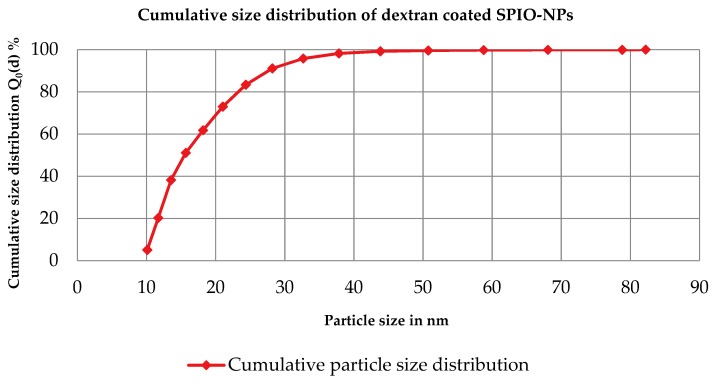
Cumulative size distribution Q_0_(d) for magnetite (z-average = 40 nm, PDI) = 0.37, and median particle size d_50, 0_ = 16 nm).

**Figure 2:**
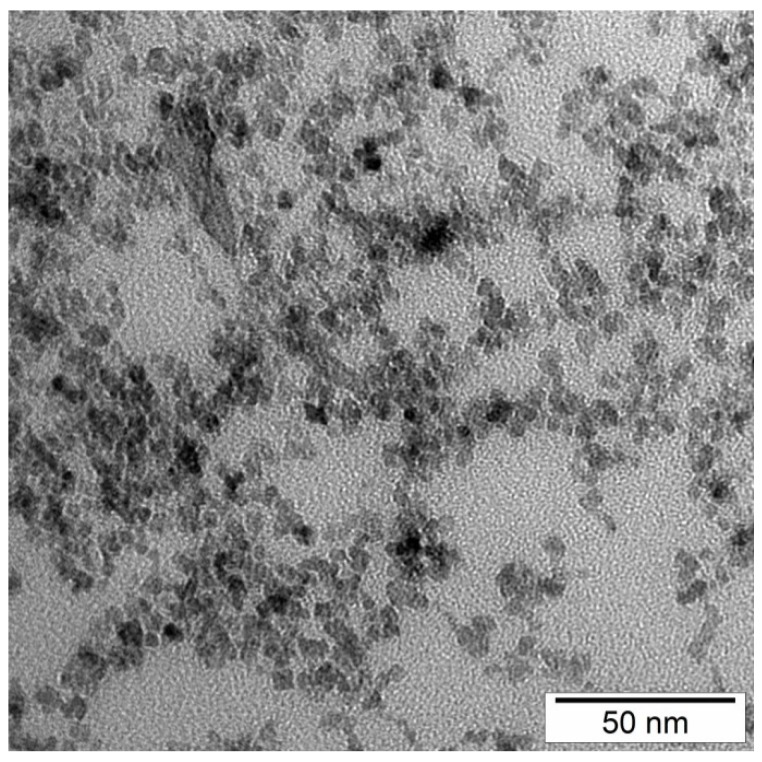
Transmission electron microscopy (TEM) of superparamagnetic iron oxide nanoparticles.

From dynamics light scattering (DLS) data, we conclude that nanoparticles of z-average 40 nm size with polydispersity index (PDI) of 0.37 were obtained in a co-precipitation reaction. Figure 1 shows the number-based distribution with some polydispersity of obtained nanoparticles. Figure 2 is an example of the transmission electron microscopy (TEM) investigation of our sample. From the TEM results, it is clear that the morphology of the nanoparticles is nearly spherical, with some agglomerates. The mean agglomerate size is 50 nm, with some primary particles of 10 to 40 nm size, which is consistent with our DLS results. Also, the nanoparticles’ size distribution from both DLS and TEM is very similar.

### 2.2. In Vitro Toxicity of Coated and Fluorescent Iron Oxide Nanoparticles by MTT Assay

**Figure 3:**
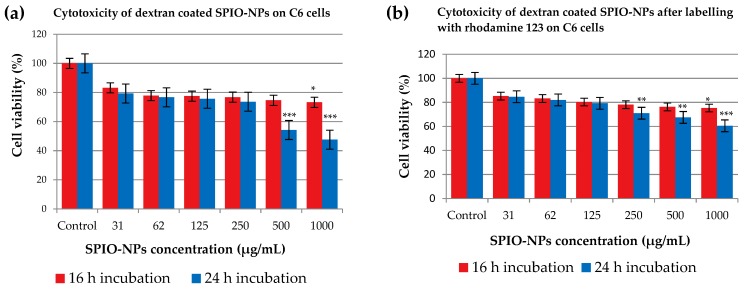
The effects of dextran 70,000-SPIO-NPs (**a**), and dextran 70,000-SPIO-NPs labelled with rhodamine 123 (**b**) on the viability of C6 glioma cells, determined by MTT assay. Statistical analysis revealed that 16 h incubation with 1000 µg/mL dextran 70,000-coated SPIO-NPs significantly reduced the cell viability as compared to control (* *p* < 0.05). In the case of 24 h of NPs incubation, 500 and 1000 µg/mL induced the cell viability significantly (each *** *p* < 0.001). For the rhodamine 123-labelled dextran 70,000-coated SPIO-NPs, results were similar: cell viability was only significantly reduced at a NPs concentration of 1000 µg/mL when incubation time was 16 h (* *p* < 0.05). However, when NPs remained for 24 h in the cell culture, 1000, 500, and 250 µg/mL concentrations induced a significantly reduced cell viability, as compared to control (** *p* < 0.01; *** *p* < 0.001). For each group, 5 wells per plate were treated identically, and each experiment was performed with at least 3 plates.

C6 glioma cells were exposed to dextran 70,000-coated SPIO-NPs, and viability was quantified using the MTT assay (Figure 3a). At lower concentration of 31 µg/mL, the cell viability rate for 16 h and 24 h incubation was 83.19% and 79.30%, respectively. At a higher concentration of 250 µg/mL, the cell viability rate of 76.76% was recorded at 16 h incubation, and after 24 h incubation, the cell viability rate was 73.65%. Interestingly, at further higher concentrations of 500 and 1000 µg/mL, the cell viability was more impaired in the 24 h incubation group than in the 16 h incubation group. At the highest concentration of 1000 µg/mL, the cell viability was 73.24% at 16 h incubation but a viability rate of 47.58% was observed at this highest concentration for the 24 h incubation group. When compared to control, 16 h incubations with the highest concentration resulted in a significant reduction in viability. When NPs were left for 24 h in the culture, the 500 and 1000 µg/mL dosages induced significant impairment.

**Figure 4:**
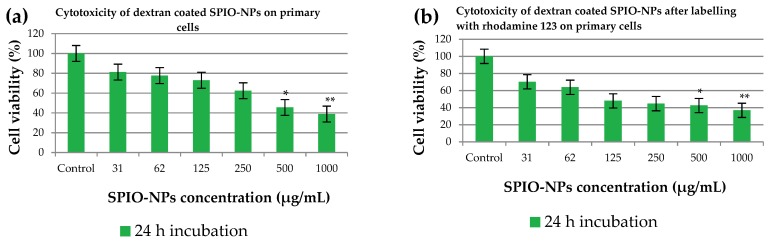
The effects of dextran 70,000-SPIO-NPs (**a**), and dextran 70,000-SPIO-NPs labelled with rhodamine 123 (**b**) on viability of primary cells, determined by MTT assay. Statistical analysis revealed that both SPIO-NPs variants induce significant reduction in viability at 500 and 1000 µg/mL. For each group, 5 wells per plate were treated identically, and each experiment was performed with 3 plates, * *p* < 0.05; ** *p* < 0.01 as compared to control.

When primary neural cells were treated with the dextran 70,000-coated SPIO-NPs for 24 h, the results were in accordance with the experiments using C6 cells (Figure 4a). At the concentration of 31 µg/mL, the neural viability was 81.18%, which is similar to the result in C6 cells. There was no significant difference observed at higher doses of 62 µg/mL and 125 µg/mL SPIO-NPs. However, further increasing the SPIO-NPs dosage to 500 or 1000 µg/mL induced a significant decrease in cell viability to 45.53% (* *p* < 0.05) or 38.82% (** *p* < 0.01), respectively. Publications about in vivo SPIO application indicate that the concentrations applied in our in vitro experiments are approximately in the range used for in vivo treatments. Elias et al. [27] injected 3.75 mg Fe/kg intravenously in rodents. Assuming that blood volume is approximately 7% of body weight [28], this translates to 50–55 µg Fe/mL blood volume.

When dextran 70,000-coated SPIO-NPs were labelled with fluorescent dye rhodamine 123 (Figure 3b), the cell viability was also very high for both incubation times, and for a broad range of nanoparticles concentrations. At the highest concentrations of 500 and 1000 µg/mL, we again detected a difference between 16 and 24 h incubation times, the cells being less viable after 24 h. Our findings were confirmed when the same rhodamine 123-labelled SPIO-NPs were incubated for 24 h with the primary neural cells (Figure 4b). At lower concentration of SPIO-NPs, our nanoparticles are non-toxic, but toxic effects appear at higher concentrations (1000 µg/mL NPs: ** *p* < 0.01; 500 µg/mL NPs: * *p* < 0.05). From this finding, it can be concluded that rhodamine 123, when attached to dextran 70,000-coated SPIO-NPs, does not substantially increase the toxicity of the nanosystems. These findings strengthen our hypothesis that these nanoparticles at low concentration are non-toxic for both C6 cells and neural primary cells.

SPIO-NPs are considered biocompatible and effective for many important pharmaceutical applications; but for properly designing these SPIO-NPs, many side effects must be considered for such uses. An important criterion that must be taken into account is the stability of synthesized SPIO-NPs in biological fluids with their physiological pH. To solve this issue, SPIO-NPs must be coated with the biocompatible surfactants, which not only prevent SPIO-NPs from agglomeration, but also increase their biocompatibility [29]. Dextran-coated SPIO-NPs also have higher cellular uptake [29]. During MTT assay for in vitro cytotoxicity, the amount of formazan produced due to mitochondrial activity is directly proportional to the number of living or viable cells, as detected by a spectrophotometer with light absorption [30]. The SPIO-NPs are safe enough and non-toxic when the exposed cells’ viability is approximately 80% or higher [30].

Obtained results suggest that coated SPIO-NPs show virtually no toxic effect at low concentrations and short incubation times. The coating on NPs also influence toxicity; there have already been reports on the surface modifications of various NPs with different masking agents that have significant chemical reactivity, and therefore, may sometimes induce acute toxic effects [31]. Our synthesized SPIO-NPs are very small in size, and due to this small size, nanoparticles interact with cells and other subcellular structures. Information for this interaction mechanism is still lacking in the existing literature [32]. Cytotoxicity of different iron oxide nanoparticles has already been reported in various studies. One common investigation in these studies is that the low concentration (~50 µg/mL) produces minimum toxicity, or is sometimes non-toxic [32]. For our nanoparticles, which are in a superparamagnetic regime, we show that, in a C6 cell line, there is low toxicity up to 250 µg/mL for a short incubation time, and also after a longer incubation of 24 h. As expected, primary hippocampal cells were more sensitive, but also showed only low toxicity at lower concentrations of up to 62 µg/mL. It has been shown that MNPs show toxicity due to reactive oxygen species (ROS) production. ROS have the short lifetime of intermediate compounds, and the compounds have a free unpaired electron in their outermost shell; thus, to have a stable structure, they obtain an electron from their adjacent molecules, which themselves becomes unstable, and this continues as a chain reaction [33]. It can be hypothesized that ROS generation may also be a mechanism underlying the reduced viability after incubation with high doses of SPIO-NPs (500 and 1000 µg/mL). However, as we see significantly impaired viability mainly after long incubations of 24 h, we most probably do not induce necrotic cell death with our NPs, as this would likely be detectable after a few hours. Hence, in the case of ROS-induced damage, it may be that only low amounts are generated, which may activate pathways of delayed cell death like apoptosis, as suggested by Bae et al. [34].

However, it has been shown that the reduced generation of formazan does not necessarily equal cell death, but that there can be a reversible decrease in enzymatic cell activity. Therefore, it is also possible that in our experiments, especially with low concentrations of dextran 70,000-coated and labelled SPIO-NPs, no cell death was induced at all, but only a reduction in metabolic activity.

### 2.3. In Vitro, In Vivo, and Ex Vivo Studies of Uptake of Coated and Fluorescent Iron Oxide Nanoparticles

**Figure 5:**
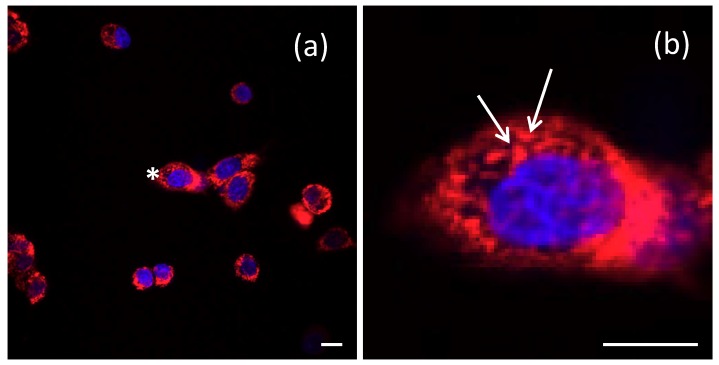
Intracellular localization of dextran 70,000-coated SPIO-NPs labelled with rhodamine 123, in C6 cells after 24 h of incubation, is shown by confocal microscopic images (**a**). The image on the right (**b**) zooms into the cell labeled with an asterisk in (**a**) to illustrate the dotted labelling (arrows), which presumably indicates endosomal–lysosomal uptake. Nuclei stained with Hoechst 33342 in blue. Scale bars 20 µm.

**Figure 6:**
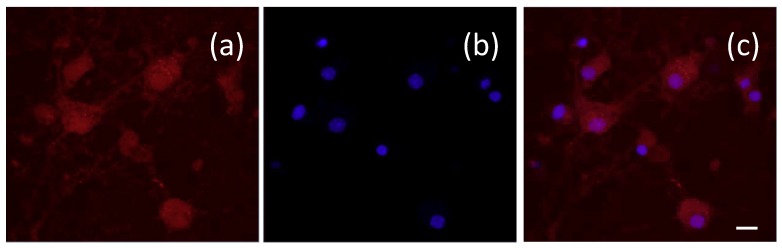
Intracellular localization of dextran 70,000-coated SPIO-NPs labelled with rhodamine 123 in primary hippocampal neurons after 24 h of incubation. The rhodamine 123 fluorescence shows similar distribution as in the C6 cells: (**a**) rhodamine 123 labelling, (**b**) blue staining of nuclei with Hoechst 33342, (**c**) merge of images (**a**,**b**). Scale bar 20 µm.

The results indicate that dextran 70,000-coated SPIO-NPs are taken up by the cells in these cell culture models after 24 h of incubation with a concentration of 500 µg/mL. A double-labelling performed with Hoechst 33342—which selectively stains the nucleus of cells—revealed that the SPIO-NPs are accumulating in the cell soma, but mostly do not enter the nucleus of C6 cells. In primary neural cell cultures, the somatic distribution is virtually the same as in C6 cells. However, there appears to be some staining also in the nucleus, which may indicate damage-induced leakiness. In the soma of both cell types, the fluorescent signal—and therefore presumably the SPIO-NPs—are distributed in a dotted pattern (Figure 5b; arrows) which suggests an endosomal–lysosomal uptake. A similar intracellular localization of rhodamine 123 labelled dextran 70,000-coated SPIO-NPs in primary hippocampal neurons is shown in Figure 6 after double labelling with Hoechst 33342.

**Figure 7:**
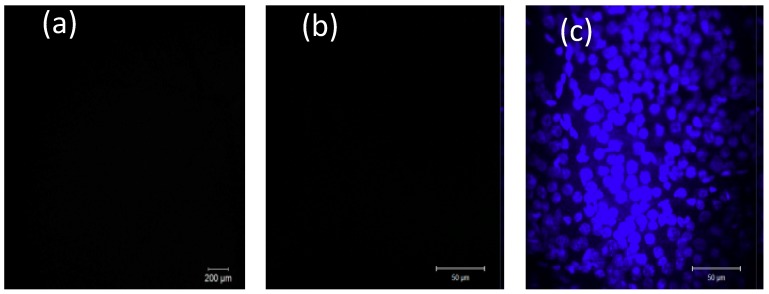
Imaging of retina in control animal experiments where rats received only phosphate buffered saline intravitreally. As expected, no fluorescence can be detected either in vivo (ICON) (**a**) or in the ex vivo retina whole mount preparation (**b**) (red channel for rhodamine 123 detection). (**c**) The retina whole mount, nuclei staining by Hoechst 33342. Scale bars: (**a**) 200 µm; (**b**,**c**) 50 µm.

**Figure 8:**
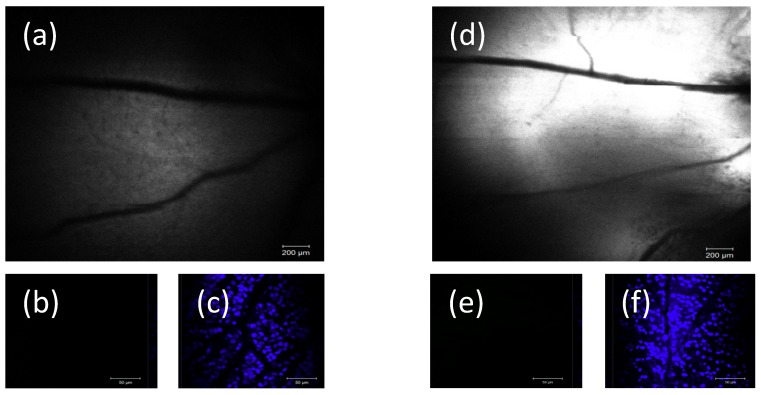
Left and right panels, (**a**,**d**), example of two different animals injected with different amounts of SPIO-NPs. Two days after intravitreal injection of rhodamine 123-labelled SPIO-NPs, the in vivo images of the retina (ICON) clearly illustrate a dose-dependent fluorescent signal (monocolor image, rhodamine 123 fluorescence). A low concentration of NPs (31 µg/mL) was injected in (**a**) and a higher concentration of NPs (500 µg/mL) was injected in (**d**). Note a minor misalignment in the upper right quadrant of image (**d**), due to minor movement of the rat during scanning. However, no rhodamine 123 fluorescence could be detected in retina whole mount preparation from the animals (**b**,**e**). (**c**,**f**) images are from the same areas as in (**a**,**e**) with Hoechst 33342 staining. Scale bars: (**a**,**d**) 200 µm; (**b**,**c**,**e**,**f**) 50 µm.

Using dissociated cell cultures, the basic ability of SPIO-NPs to cross the cellular membrane and accumulate inside the cell was demonstrated. However, in an in vivo situation, additional factors can influence the uptake kinetics, as compared to the situation of cells in vitro. Therefore, we injected SPIO-NPs into the eyes’ vitreous body of live animals. The vitreous body and the retina can be seen as a natural in vivo “dish” of live and intact brain parenchyma which, most importantly, is accessible to microscopic imaging through the eye’s optical system (cornea, lens). This enabled us to visualize, by in vivo confocal neuroimaging (ICON), the subsequent distribution and possible uptake of the SPIO-NPs into retina tissue of living rats. Two days after injection, ICON of the retina was performed. The images indicate a dose-dependent, diffuse distribution of the nanoparticles at the retina by focusing at the upper level of the retina-layers (inner region)—with the major retinal arteries as landmarks. Whereas in control animals, which had received only vehicle injection, no fluorescence was detected (Figure 7a), we revealed a strong fluorescent signal in rats injected with the high dose SPIO-NPs (Figure 8d). In animals injected with a low dose of labelled SPIO-NPs, we saw a weak, diffuse signal (Figure 8a).

Interestingly, ex vivo, after preparation of the retina whole mount, we did not detect any fluorescent signal of rhodamine 123 anymore. Since the whole mount had been incubated with Hoechst 33342, as expected, cellular nuclei showed up in bright blue (Figure 7c and Figure 8c,f). These results indicate that, after intravitreal injection, the SPIO-NPs attach to the retina surface, as shown by ICON, however, probably not with direct contact to neural cells, but only to the inner limiting membrane and basal lamina which cover the retina tissue as a boundary to the vitreous humour. Our results suggested that the particles are not taken up by the retinal cells or extracellular matrix after intravitreal injection. When being attached to the inner limiting membrane, the SPIO-NPs can be washed away during the preparation procedures of the retina whole mount and, therefore, the nanoparticles with the rhodamine 123 signals disappeared, and are not detectable anymore during subsequent imaging of the whole mount. In the case of cellular uptake, we would expect rhodamine 123 labelling also in the whole mount preparation.

This contrast of in vivo and ex vivo results leads to the assumption that the inner limiting boundary is not endowed with the same uptake mechanisms as the lipid bilayer barrier around cells. Interestingly, in contrast to our relatively small SPIO nanoformulations (40 nm) we recently show that polymeric-based NPs, with an average diameter of 145 nm, could deliver their drug load across the inner limiting membrane into retinal cells after intravitreal injection [35]. Therefore, it seems that there exists specific biological boundaries and a sheath which safeguard cells against unwanted uptake of SPIO-NPs.

## 3. Materials and Methods

### 3.1. Materials

Ferrous chloride (FeCl_2_·4H_2_O) and ferric chloride (FeCl_3_·6H_2_O) were purchased from Carl Roth (Karlsruhe, Germany). Dextran 70,000 and fluorescent dye (rhodamine 123) were purchased from AppliChem (Darmstadt, Germany). For the cell culture tests, thiazolyl blue tetrazolium bromide and dimethyl sulfoxide were obtained from Sigma Aldrich (Taufkirchen, Germany), and phosphate buffered saline (pH 7.2) was used from Thermo Fischer Scientific (Waltham, MA, USA). Hoechst 33342 was purchased from Cayman Chemicals (Hamburg, Germany). All other chemicals were purchased from Sigma Aldrich (Germany), Biochrom (Berlin, Germany) and Thermo Fischer Scientific (USA). Stock solutions were prepared just prior to their utilization.

All chemicals were used as received without further purification.

### 3.2. Synthesis of Superparamagnetic Iron Oxide Nanoparticles

We used a co-precipitation method to synthesize the iron oxide (Fe_3_O_4_) nanoparticles which is based on Massart’s method [36,37], due to its simplicity and suitability for a variety of metal oxides [38]. To optimize the precipitation process, various influencing parameters were controlled. The control of size, shape, and nanoparticle properties during the co-precipitation method strongly depends on the types of salts, pH, temperature, supersaturation, mixing velocity (stirring), and Fe^3+^/Fe^2+^ molar ratio [39,40,41].

Typically, 2.2 g of FeCl_3_·6H_2_O and 0.8 g of FeCl_2_·4H_2_O with 2:1 molar ratio were dissolved in deionized (DI) water in a three-neck flask under vigorous stirring. Five grams of surfactant (dextran) were solubilized separately in deionized (DI) water in a beaker, and then added into the three-neck reaction flask. The reaction temperature was adjusted to 40 °C, to which 25% NH_3_ solution as a precipitating medium was injected into the glass reactor under stirring. After 1 h reaction under the same conditions, a dark black solution was obtained, and stored in clean polypropylene plastic bottles. For labeling with a fluorescent marker, a stoichiometric amount of fluorescence dye was dissolved in DI water, and then a stoichiometric amount of iron oxide nanoparticles was introduced, followed by electronic shaking. The nanoparticles syntheses were probe-sonicated for 10 min. The overall reaction during the co-precipitation method is

2Fe^3+^ + Fe^2+^ +8OH^−^ → 2Fe(OH)_3_·Fe(OH)_2_↓→ Fe_3_O_4_↓ + 4H_2_O.

Nanoparticle size was controlled/adjusted through a suitable choice of process and apparatus parameters during the synthesis.

### 3.3. Determination of Particle Size, Zeta Potential, and Morphology

Particle size, particle size distribution, and zeta potential were measured by Malvern zetasizer (PCS nano ZS, UK) that is based on photon correlation spectroscopy. Transmission electron microscope (TEM), with high-resolution Tecnai F20 (FEI Company, Hillsboro, OR, USA) with high-angle annular dark-field imaging (HAADF) and annular dark-field imaging (ADF) detectors, was used to study the morphology of the synthesized superparamagnetic iron oxide nanoparticles. Specimens were prepared by drying the liquid sample drops on a glass grid for TEM investigations.

### 3.4. Cell Cultures of C6 Glioma Cells

The rat glioma-derived C6 cells were grown under standard conditions in a 82.5% Ham’s F-12K medium, supplemented with 15% horse serum, 2.5% fetal bovine serum (FBS), and 1% antibiotics (pencillin–streptomycin solution). The cells were incubated at 37 °C in a 5% CO_2_ humidified atmosphere. For the MTT assay, the cells were plated into a 96-well plate. After 24–48 h of incubation, plates were taken to perform the MTT assay.

### 3.5. Culturing of Neural Primary Cells

Primary hippocampal cells were dissociated from embryonic mice on embryonic day 18. To prepare the primary neural cultures, pregnant C57/BL6J mice were sacrificed by cervical dislocation. Embryos were decapitated, and the hippocampi isolated from brains under sterile conditions and dissected. Enzymatic digestion by 0.25% trypsin/EDTA solution was followed by separation of the cells in serum-containing medium via trituration (10 passes, 1 mL pipette). The primary cells were seeded onto coated (with 50 µL of 0.1% polylysine) 96-well plates in 200 µL Dulbecco’s modified eagle’s medium (DMEM) per well (supplemented with 1% penicillin-streptomycin and 1% L-glutamine and 2% B27). After incubation for 2 h at 37 °C/5% CO_2_, the medium was exchanged with the pre-warmed neurobasal medium including 1% penicillin–streptomycin, 1% l-glutamine, and 2% B27. One half of the medium was changed every 2 days. The cells—predominantly neurons and astrocytes—were incubated at 37 °C in 5% CO_2_ humidified atmosphere for 14 days before administration of nanoparticles.

### 3.6. MTT Assay

C6 cells and primary neural cells grown in a 96-well plate were exposed to dextran 70,000-coated iron oxide nanoparticles (with and without fluorescent dye) at different concentrations (31, 62, 125, 250, 500, and 1000 µg/mL), and then incubated for 16 and 24 h. Primary cells were incubated for 24 h. After incubation, a thorough washing was done with 100 µL phosphate buffered saline (pH = 7.2). After washing, the phosphate buffered saline was completely removed, followed by the addition of cell culture medium. MTT solution (3-(4,5-dimethylthiazol-2-yl)-2,5-diphenyltetrazolium bromide), 10 µL reagent (5 mg/mL), was added per plate and then, again, the plates were incubated for another 4 h in an incubator. After 4 h incubation, the solution was removed from each well, and 100 µL of dimethyl sulfoxide (DMSO) was added to each well, followed by shaking of the plate at 5–6 rpm for approximately 10 min. When the crystals were dissolved in the solution, it turned from yellow to violet. DMSO destroyed the cells and released the formazan, which was detected by optical density (OD) value at 560 nm, measured using a plate reader (spectrophotometry Opsys MR-Dynex, West Sussex, UK).

For confocal imaging, C6 glioma and hippocampal cells were treated with SPIO-NPs for one day and after adding Hoechst 33342 (in medium), and cells were incubated for at least 40 min. Just before imaging, the medium was removed and replaced with fresh phosphate buffered saline. A magnification 50× was chosen for all images, and the laser lines used for imaging were 405 nm for Hoechst and 514 nm for rhodamine 123.

### 3.7. Animal Model

For in vivo uptake study, 12- to 13-week-old male Wistar rats were used. They were maintained in group cages at 12 h light/12 h dark cycles under 24–26 °C and 50–60% humidified conditions. All rats had ad libitum access to their food and water; however, one day before experiment, food was removed to facilitate narcosis. Rats were weighed and put under a heating lamp for 5 min before narcosis injection. After intraperitoneal narcosis injections (5 mL/kg body weight of a mixture of 2 mL ketamine 100 mg/mL, 3 mL medetomidine 1.0 mg/mL, and 15 mL of 0.9% saline), the rats were randomly divided into three groups with 3 animals per group: a solution of 3 µL of dextran-coated SPIO-NPs labelled with rhodamine 123 at 2 different concentrations, or vehicle, were locally injected into the eyes’ vitreous body of the anaesthetized rats (concentrations of 31 and 500 µg/mL). Two days afterwards, in vivo confocal neuroimaging was performed, and subsequently also ex vivo (whole mounts) imaged. For all procedures, ethical approval was obtained according to the requirement of the German National Act on the use of Experimental animals (Ethic committee Referat Verbraucherschutz, Veterinärangelegenheiten; Landesverwaltungsamt Sachsen-Anhalt, Halle, Germany; AZ42502-2-1283; 12 January 2015; amendment for extension 20 October 2017).

#### 3.7.1. In Vivo Confocal Neuroimaging (ICON)

The rats were fixed on a purpose-built stage underneath a standard confocal laser scanning microscope, with a large probe space and a long working distance objective lens. To prevent drying of the eye of anesthetized rats, an optical gel was applied. The eye was positioned directly underneath the 5× objective lens of a Zeiss LSM 880 (Jena, Germany) and a −80 diopter plan concave lens was placed directly onto the surface of the cornea, to adjust the path of the laser rays to the rat’s eye. Images (ICON and ex vivo) were taken two days after the intravitreal injection of nanoparticles.

#### 3.7.2. Whole Mount (Ex Vivo)

The rats were decapitated under deep ketamine/medetomidine narcosis. Eyes were enucleated and placed in cold HEPES-buffered Ca^2+^-free solution containing 135 mM, NaCl, 5 mM NaOH, 2.5 mM KCl, 7 mM MgCl_2_, 10 mM HEPES, 10 mM glucose; pH 7.4. The excess of connective tissues and muscles were removed. The cornea was removed by cutting on the rim with the sclera. The lens and most of vitreous were removed, and the retina was separated from the pigment epithelium. Residuals of vitreous were removed either by small forceps or a wooden toothpick. Small cuts (3–4) from the edge direction to optic nerve were performed, and the retina was flattened onto a hydrophilic polytetrafluoroethylene (PTFE) membrane (0.4 µm pore). To fix the retina, a 4% paraformaldehyde solution was applied for 10 min.

The retina was washed with HEPES ((4-(2-hydroxyethyl)-1-piperazineethanesulfonic acid)) buffer, and then Hoechst 33342 (0.005 mg/mL) was added to counterstain nuclei. The imaging was done using Zeiss LSM 880 confocal microscope with 50× magnification.

### 3.8. Statistics

Statistical analysis was performed with the data presented in Figure 3 and Figure 4. Due to the relatively low sample size, no normality tests were performed, and group comparison was implemented using the non-parametric equivalent of ANOVA, the Kruskal–Wallis test, followed by Dunn’s post hoc tests for pairwise comparisons with the control group.

## 4. Conclusions

Dextran 70,000-coated SPIO-NPs were investigated in C6 glioma cells and primary neural cells, regarding their internalization and toxicity, in a concentration-dependent manner. The toxicity profile on C6 cells shows that coated and fluorescent SPIO-NPs are non-toxic at low concentrations, and also at short incubation times. The threshold limit is 250 µg/mL. In addition, hippocampal neurons were exposed to SPIO-NPs for 24 h, which had no detectable toxic effects when the NP concentrations were 62 µg/mL or lower. This is an important aspect for translational studies, as SPIO-NP toxicity on cancer cells (glioma cells) may not raise too much concern, but a massive damage of neurons may be a serious safety issue. As previously described, nanoparticles’ toxicity depends on numerous physicochemical properties. Reactive oxygen species (ROS) have been described as a cause of nanoparticle toxicity, and this becomes acute in smaller sized SPIO-NPs, as compared to the large-sized SPIO-NPs. Our coated nanoparticles are very small in size (around 40 nm), however, are well tolerable. For our in vitro model, cellular uptake was quite high and we demonstrated accumulation of the fluorescent SPIO-NPs in the soma. In vivo, after intravitreal injection, our NPs were, most likely, attached to the inner limiting membrane, and this barrier seems to prevent the particles’ uptake by neural cells. This indicates that basal membranes can be a barrier for SPIO-NPs. Overall, the association of in vitro cellular uptake and low toxicity elucidates the biocompatibility of our SPIO-NPs. Hence, these SPIO-NPs may be useful in many pharmaceutical applications, especially diagnostic and drug delivery.

## Abbreviations

ADF	annular dark-field
BBB	blood–brain barrier
DI	deionize
DLS	dynamic light scattering
DMEM	dulbecco’s modified eagle’s medium
DMSO	dimethyl sulfoxide
FBS	fetal bovine serum
FDA	federal drug association
HAADF	high-angle annular dark-field
ICON	*in vivo* confocal neuroimaging
LCD	liquid-crystal display
LDH	lactate dehydrogenase
MNPs	magnetic nanoparticles
MRI	magnetic resonance imaging
NPs	nanoparticles
OD	optical density
PDI	polydispersity index
PTFE	polytetrafluoroethylene
ROS	reactive oxygen species
SPIO-NPs	superparamagnetic iron oxide nanoparticles
TEM	transmission electron microscopy
UV	ultraviolet
